# Real world clinical practice patterns in nocturnal GERD management: insights from nationwide RELIEF survey

**DOI:** 10.1186/s12876-026-05084-x

**Published:** 2026-07-23

**Authors:** Sandip Pal, G. Manoharan, Prashant Bhandarkar, P Manohar Reddy, Atul Shende, R. S. Arun, Balaji Laxminarayana Shetty B, Saptarshi Bishnu, Sanjeeb Roy, Sucheta Mehta, Namita Ghag, Apurva Jawdekar, Aniket Vaidya

**Affiliations:** 1 Senior Consultant Gastroenterologist and Hepatologist, Narayana Health, Kolkata, India; 2 Consultant Hepatologist and Gastroenterologist, Gastro Care Clinic, Chennai, India; 3 Consultant Gastroenterologist, Gastroenterology and Endoscopy Centre, Dhantoli, Nagpur, India; 4 Consultant Hepatologist and Gastroenterologist, Asian Institute of Gastroenterology, Hyderabad, India; 5 Department of Medicine, M.Y. Hospital, Indore, India; 6 Senior Consultant and Head, Institute of Gastroenterology and Liver Diseases, Chennai, India; 7 Consultant Gastroenterologist, Manipal Hospital, Bengaluru, India; 8 Consultant Hepatologist and Gastroenterologist, Apollo Hospital, Burdwan, West Bengal India; 9 Fellow in Advanced GI Endoscopy, Narayana Health, Kolkata, India; 10 Cipla Ltd, Mumbai, India

**Keywords:** Nocturnal GERD, Proton pump inhibitors, Esomeprazole, Health-related quality of life, Heartburn, Sleep disturbance

## Abstract

**Background and objectives:**

Nocturnal symptoms of GERD interfere with daytime functioning, work, and social activities, thereby negatively affecting health-related quality of life. Therefore, the present survey aimed to evaluate the clinical practice patterns regarding the management of nocturnal GERD in India.

**Methods:**

A digital questionnaire-based nationwide RELIEF survey was conducted among health care practitioners (HCPs) with gastroenterology practice across India. Physicians voluntarily responded to the survey questionnaire consisting of 24 questions based on their clinical experience. Following the collection of completed questionnaires, data were reviewed and analyzed.

**Results:**

Of the 500 HCPs who were approached, 498 completed the study. GERD contributed to > 30% of patient burden every month. According to 25.30% of HCPs, 21–30% of patients with GERD complain of nocturnal symptoms. The most common risk factors in patients with nocturnal GERD were late-night meals (67.67%), followed by smoking (51%), BMI > 25 kg/m^2^ (50.20%), and a high-fat diet (50.40%). Most HCPs (83%) relied on symptoms and medical history to diagnose nocturnal GERD. A single-dose proton pump inhibitor (PPI) regimen was the most preferred treatment, reported by 31.53% of HCPs, with esomeprazole preferred by 54% of physicians. Efficacy (58.03%), minimum or no drug-drug interactions (29.92%), and cost (22.49%) are the important aspects for choosing a PPI. HCPs (63.18%) opted for 4–8 weeks of PPI therapy to achieve resolution of nocturnal GERD symptoms, followed by 3–6 months of maintenance therapy (57.95%). Common causes of non-adherence to PPIs, as reported by 80.72% of HCPs, were stopping treatment once symptoms improved (53.01%), cost (39.56%), and incomplete disease awareness (29.92%). Most of the HCPs (42.77%) reported no side effects with the use of PPIs.

**Conclusion:**

The survey highlighted key insights into managing nocturnal GERD in India, with nearly one-fourth of GERD patients reporting nocturnal symptoms like heartburn and regurgitation, emphasizing the need for lifestyle modifications and suitable pharmacological interventions. The management strategy primarily includes PPI therapy, with esomeprazole being the preferred choice for nocturnal GERD.

**Supplementary Information:**

The online version contains supplementary material available at https://doi.org/10.1186/s12876-026-05084-x.

## Introduction

Gastroesophageal reflux disease (GERD) is a prevalent gastrointestinal condition among patients visiting primary care physicians, gastroenterologists, and others, with a prevalence ranging from 5% to 28.5% in the Indian population [1]. The typical symptoms of GERD include heartburn and regurgitation; however, patients may also experience other issues like nausea, epigastric pain, dysphagia, chronic cough, and sleep disturbances [2]. Though there is no standardized definition available for nocturnal GERD, large surveys have identified key symptoms used to define the condition including: (1) night time awakening due to heartburn; (2) being awakened at night by coughing or choking caused by fluid, an acidic or bitter taste, or food in the throat; (3) heartburn that occurs while lying down at night; and (4) waking up in the morning with heartburn and/or acid regurgitation. Nocturnal gastroesophageal reflux is the cause of sleep disruptions, frequently experienced by up to 25% of patients with GERD [3]. Patients with nocturnal reflux often report difficulty falling asleep, fragmented sleep patterns, and waking up in the middle of the night. Approximately 80% of individuals with GERD report experiencing nocturnal symptoms [4, 5], which disrupt daytime functioning, occupational performance, and social activities, ultimately resulting in a deterioration in health-related quality of life [4–6]. Lifestyle modifications, such as elevating the head of the bed (6–8 inches) or sleeping on a wedge, can be effective in improving nocturnal GERD symptoms and reducing nocturnal esophageal acid exposure [7–9]. Patients might also be advised to avoid sleeping on their right side and to refrain from eating meals 2–3 h before bedtime [10, 11]. Despite these measures, lifestyle changes often do not provide sufficient relief.

Pharmacological treatments can play a critical role in managing nocturnal GERD. Proton pump inhibitors (PPIs) are highly effective for the treatment of nocturnal reflux symptoms, showing reductions in reflux-related sleep disturbances and improvements in sleep quality and work productivity [11]. Although once-daily PPI treatment alleviates night-time heartburn, it does not eliminate it completely [12]. The use of bedtime H2 receptor antagonists (H2RAs) may be beneficial if dosed on an as-needed basis for patients with nocturnal symptoms, and for those with objective evidence of nocturnal acid reflux on pH monitoring despite PPI treatment. However, the effect of H2RAs is temporary, and their clinical significance on esophageal reflux has been debated [13, 14].

Despite the common occurrence of nocturnal GERD and its significant impact on quality of life, there is a lack of understanding about the current knowledge and clinical practices among Indian physicians regarding this condition. Moreover, limited data are available regarding the management of nocturnal GERD in the Indian setting. Therefore, the present survey aimed to evaluate the clinical practice patterns regarding the management of nocturnal GERD in India.

## Materials and methods

### Study design

This digital questionnaire-based nationwide RELIEF survey was conducted from August 2023 to January 2024 in India. The survey exclusively involved the participation of Indian healthcare practitioners (HCPs) with gastroenterology practice. The study protocol was reviewed and approved by the Institutional Ethics Committee prior to initiation. All procedures were conducted in accordance with the Declaration of Helsinki and applicable regulatory guidelines. The survey protocol, consent form, and questionnaire were shared digitally with the HCPs. After providing consent, participating HCPs were requested to complete all questions in the survey based on their clinical experience.

### Participants

The healthcare professionals were selected randomly across India based on their specialty (Gastroenterologists/ Consulting Physicians), qualification (MBBS, MD ± MD/DM/DNB Gastroenterology), and experience (≥ 5 years of clinical practice).

### Data collection

The Real-World Clinical Practice Patterns in Nocturnal GERD Management: Insights from Nationwide Survey Questionnaire included a total of 24 questions to evaluate the clinical practice patterns regarding management of nocturnal GERD in India (Supplementary file 1). Healthcare practitioners’ responses on various aspects related to GERD, including disease burden, patient demographics, clinical practices, diagnostic methods, treatment regimens, and outcomes, were collected in the survey. Following the collection of all completed questionnaires, the data were reviewed and analyzed.

### Data analysis

Data was analyzed using Microsoft Excel 2019. Descriptive analysis was used to present the survey outcomes. Categorical variables were described as numbers and percentages.

## Results

A total of 500 HCPs were approached, of whom 498 completed the study. Most of the HCPs (43.35%) were ≤ 40 years of age and had ≤ 10 years of practice (42.05%) followed by 35% doctors who had 11–20 years of clinical practice. HCPs who participated in the survey were predominantly from Maharashtra (24.50%), followed by West Bengal (14.26%), Karnataka (9.44%), Orissa (5.82%), Uttar Pradesh (5.82%), Tamil Nadu (5.62%), and Madhya Pradesh (5.02%) (Table 1).

**Table 1:** Demographic characteristics of physicians who participated in the survey

Parameters	Number of physicians
Age (years)	[*n* = 496]
≤ 40	215 (43.35)
41–50	164 (33.06)
51–60	80 (16.13)
> 60	37 (7.46)
Years of practice	[*n* = 497]
≤ 10	209 (42.05)
11–20	174 (35.01)
21–30	72 (14.49)
31–40	30 (6.04)
41–50	12 (2.41)
Zone-wise distribution	[*n* = 498]
North zone	84 (16.87)
East zone	133 (26.71)
South zone	107 (21.49)
West zone	141 (28.31)
Central zone	33 (6.63)
Northeast zone	4 (0.80)

Data presented as n (%)

**Fig. 1:**
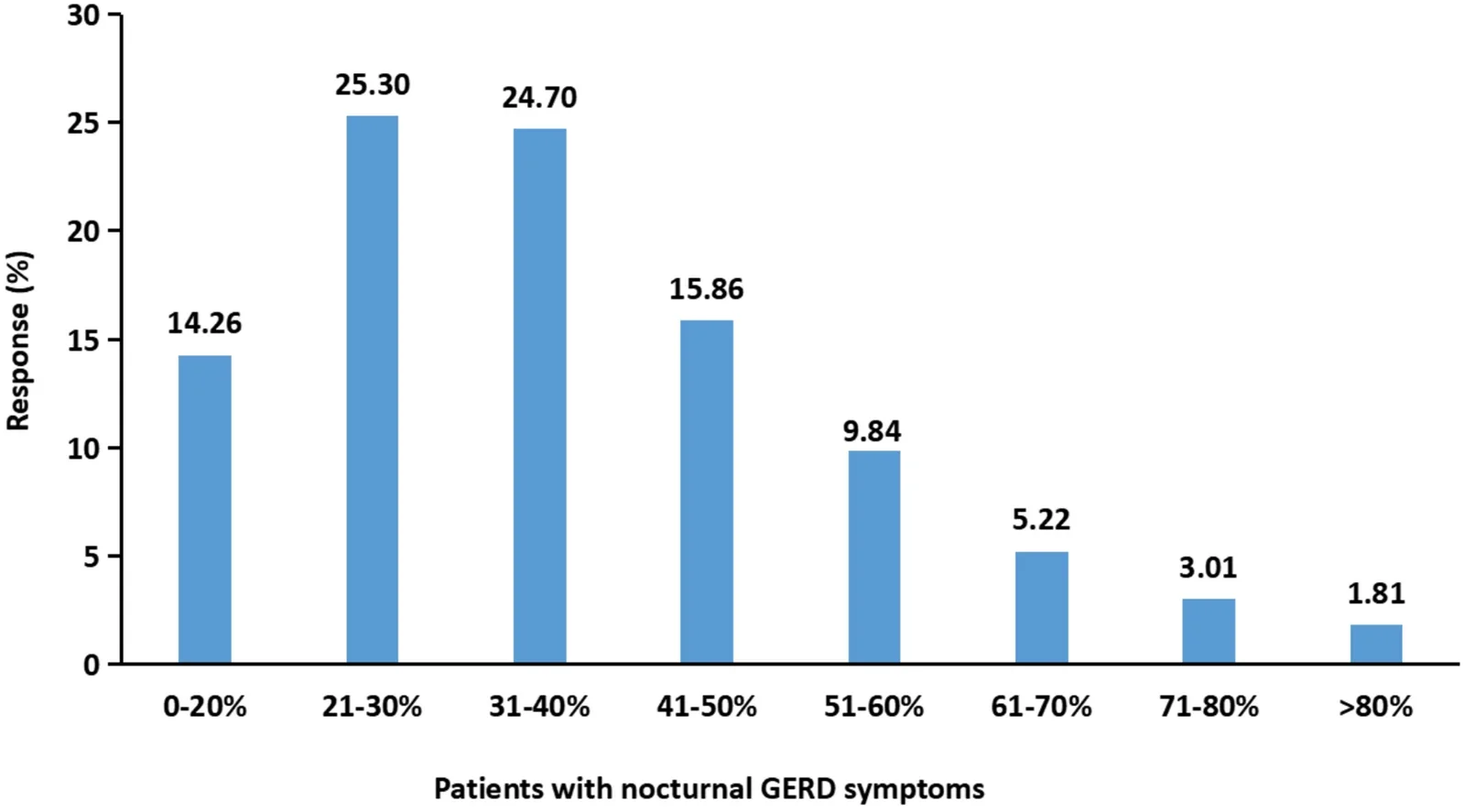
Patients with GERD reporting night-time symptoms (heartburn and regurgitation). GERD, gastroesophageal reflux disease

HCPs (25.30%) reported that 21–30% of patients with GERD complain of night-time symptoms, including heartburn and regurgitation (Fig. 1). Around 34% of HCPs encountered > 30% of patients with GERD every month. According to 41.16% of HCPs, the prevalence of nocturnal GERD was higher among males. HCPs reported that the commonly observed risk factors in patients with nocturnal GERD were late-night meals (67.67%), followed by smoking (51%), body mass index (BMI) > 25 kg/m^2^ (50.20%), and a high-fat diet (50.40%) followed by alcohol consumption (40.36%), hiatal hernia (38.35%), and long term and severe regurgitation (33.13%). The HCPs reported that nocturnal GERD symptoms were comparable in both groups i.e. patients with non-erosive reflux disease (49.40%) and erosive esophagitis (48.39%), whereas Barrett’s esophagus was reported in a minimal number of cases (2.21%). Obesity was the most common comorbidity reported by 79.52% of HCPs followed by diabetes (47.79%), sleep apnea (37.15%), depression (30.12%), and hypertension (24.50%). A list of other comorbidities reported is mentioned in Table 2.

**Table 2:** Physicians’ responses on patients with nocturnal GERD

Parameters	Number of physicians (*N* = 498)
Patients with GERD encountered per month (%)	
≥ 10	48 (9.64)
11–20	122 (24.50)
21–30	157 (31.53)
> 30	171 (34.34)
Gender-wise prevalence of nocturnal GERD	
Majority male	205 (41.16)
Majority female	166 (33.33)
Equal	126 (25.30)
Common risk factors in patients with nocturnal GERD	
Late night meals	337 (67.67)
Smoking	254 (51.00)
BMI > 25 kg/m^2^	250 (50.20)
High-fat diet	251 (50.40)
Alcohol consumption	202 (40.36)
Hiatal hernia	191 (38.35)
Long term and severe heartburn/regurgitation	165 (33.13)
Others*	16 (3.21)
Group of GERD patients most frequently associated with nocturnal symptoms	
Non-erosive reflux disease	246 (49.40)
Erosive esophagitis	241 (48.39)
Barrett’s esophagus	11 (2.21)
Common comorbidities in patients with nocturnal GERD	
Obesity	396 (79.52)
Diabetes	238 (47.79)
Sleep apnea	185 (37.15)
Depression	150 (30.12)
Hypertension	122 (24.50)
COPD	92 (18.47)
Coronary heart disease	75 (15.06)
Arthritis	58 (11.65)
Others^#^	2 (0.40)

Data presented as n (%)

*BMI *body mass index, *COPD *chronic obstructive pulmonary disease, *GERD *gastroesophageal reflux disease

*Others: Tobacco consumption (*n* = 2), shift workers (*n* = 2), sedentary habits (*n* = 2), stress (*n* = 2), anxiety (*n* = 1), empty stomach caffeine intake (*n* = 1), obstructive sleep apnea (*n* = 1), irregular meals (*n* = 1), insomnia (*n* = 1), drugs (*n* = 1), *Helicobacter pylori* infection (*n* = 1), ca. (*n* = 1)

^**#**^Others: Anemia, asthma

**Fig. 2:**
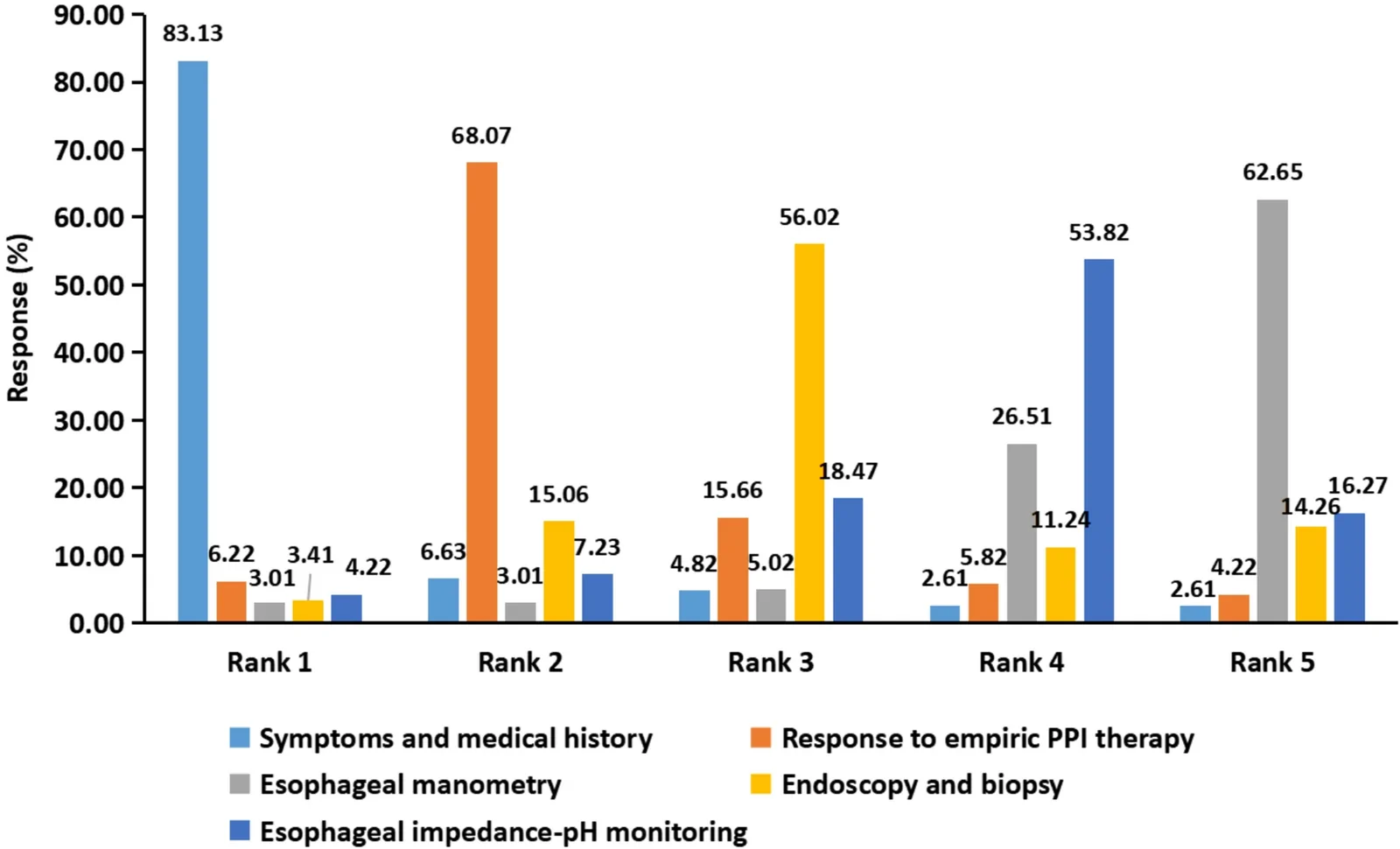
Survey response - approach to diagnosis of nocturnal GERD. GERD, gastroesophageal reflux disease; PPI, proton pump inhibitor

Most HCPs (83.13%) relied on symptoms and medical history as the primary approach to diagnose nocturnal GERD, while 68.07% relied on response to empiric PPI therapy (Fig. 2).

**Fig. 3:**
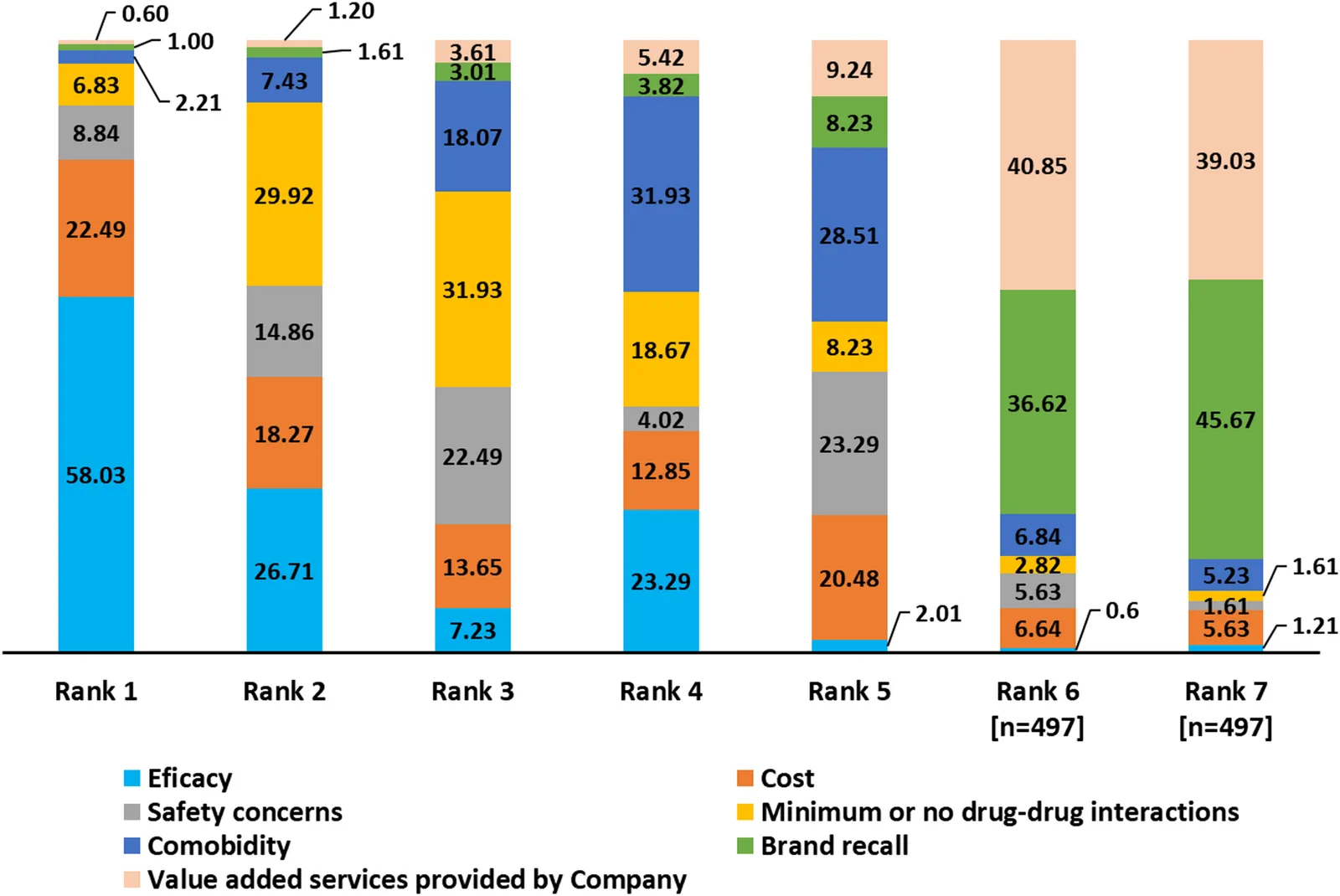
Important parameters to consider when choosing a PPI. Data shown as response (%)

**Fig. 4:**
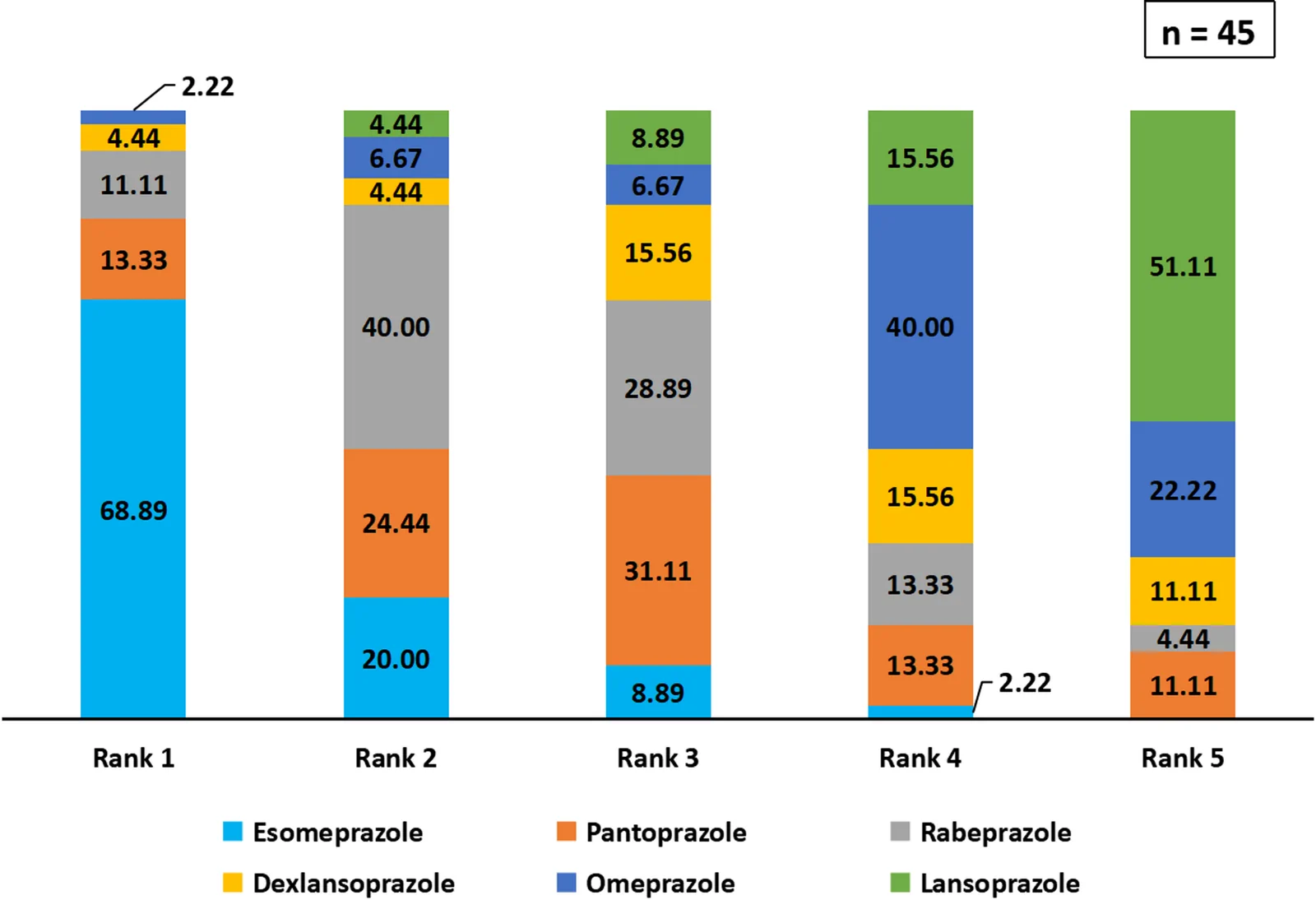
Preference of PPI for the management of nocturnal GERD. Data shown as response (%)

Efficacy (58.03%), minimum or no drug-drug interactions (29.92%) and cost (22.49%) were the important aspects for choosing a PPI (Fig. 3). Among PPIs, 68.89% of HCPs preferred esomeprazole as the first-choice PPI, while 20% of HCPs preferred it as a second-choice PPI for managing patients with nocturnal GERD (Fig. 4).

A total of 31.53% of HCPs preferred a single-dose PPI regimen (40 mg esomeprazole once daily), while 28.51% opted for a twice-daily regimen (40 mg esomeprazole twice daily). A mean duration of 4–8 weeks of PPI therapy was reported as necessary to achieve resolution of nocturnal GERD symptoms by 63.18% of HCPs. Additionally, 57.95% of HCPs reported that the mean duration of maintenance therapy required to achieve resolution of nocturnal GERD symptoms with PPIs is 3–6 months. Approximately 42% of HCPs reported that 61–80% of patients who were prescribed esomeprazole achieved symptomatic relief, while 26% reported that more than 80% of patients achieved symptomatic relief with esomeprazole. Advantages of esomeprazole relevant to the clinical response in nocturnal GERD include greater acid control (18.47%), maintenance of pH > 4 for an extended duration (17.67%), and relief of night-time heartburn /sleep disturbance (13.65%). A total of 46.18% HCPs reported a recurrence of nocturnal symptom in < 20% after completing 3–6 months of PPI treatment. According to 62.45% of HCPs, 1–5% of patients with nocturnal GERD eventually required surgical management (Table 3).

**Table 3:** Treatment regimen for patients with nocturnal GERD

Parameters	Number of physicians (*N* = 498)
Preferred treatment regimen for patients with nocturnal GERD	
Single-dose PPI (40 mg esomeprazole once daily)	157 (31.53)
Double-dose PPI (40 mg esomeprazole twice daily)	142 (28.51)
Split-dose PPI + alginate	63 (12.65)
Single-dose PPI + H2RA	60 (12.05)
Split-dose PPI (20 mg esomeprazole twice daily)	41 (8.23)
Split dose PPI + H2RA	30 (6.02)
Others	9 (1.81)
Mean duration of PPI therapy to resolve nocturnal GERD symptoms (weeks)	[*n* = 497]
< 4	105 (21.13)
4–8	314 (63.18)
> 8	78 (15.69)
Mean duration of maintenance therapy for resolution of nocturnal GERD symptoms with PPIs	[*n* = 497]
< 3 months	156 (31.39)
3–6 months	288 (57.95)
> 6 months-<1 year	42 (8.45)
≥ 1 year	11 (2.21)
Patients prescribed esomeprazole and achieved symptomatic relief (%)	[*n* = 497]
20–40	39 (7.85)
41–60	118 (23.74)
61–80	210 (42.25)
> 80	130 (26.16)
Advantages of esomeprazole that are relevant for clinical response	
Provides greater acid control	92 (18.47)
Maintains pH > 4 for 16.8 h	88 (17.67)
Relieves night -time heartburn and sleep disturbance symptoms	68 (13.65)
Improves healing rate of the esophageal mucosa	53 (10.64)
Higher systemic bioavailability	45 (9.05)
All of the above	309 (62.05)
Recurrence rate of nocturnal GERD post-PPI treatment (3–6 months), (%)	
< 20	230 (46.18)
21–40	183 (36.75)
41–60	62 (12.45)
> 60	23 (4.62)
Patients with nocturnal GERD eventually requiring surgical management (%)	
1–5	311 (62.45)
6–10	108 (21.69)
11–20	54 (10.84)
21–40	15 (3.01)
41–50	10 (2.01)

Data presented as n (%)

*GERD *gastroesophageal reflux disease, *H2RA *H2 receptor antagonists, *PPI *proton pump inhibitors

Others: PPI with prokinetics (*n* = 2), single dose PPI (*n* = 1), H2RA (*n* = 1), alginate (*n* = 1), double dose PPI + double dose H2RA (*n* = 1)

HCPs (48.80%) reported that < 20% of patients experienced recurrence and/or relapse of symptoms with non-adherence (66.67%) being the most common reason, followed by comorbidities (51.41%), inadequate dosage of medication (41.97%), incorrect diagnosis (28.11%) and other factors including poor lifestyle management (1.61%) (Table 4).

**Table 4:** Physician’s responses on patients with refractory or recurrent symptoms

Parameters	Number of physicians (*N* = 498)
Patients reporting refractory or recurrent symptoms (%)	
< 20	243 (48.80)
20–30	150 (30.12)
31–40	55 (11.04)
41–50	28 (5.62)
51–60	14 (2.81)
> 60	8 (1.61)
Common reasons for refractory or recurrent symptoms	
Non-adherence	332 (66.67)
Comorbidities	256 (51.41)
Inadequate dosage	209 (41.97)
Incorrect diagnosis	140 (28.11)
Others	8 (1.61)

Data presented as n (%)

*GERD *gastroesophageal reflux disease

Others: Smoking (*n* = 2), lost to follow-up (*n* = 1), hiatal hernia (*n* = 1), diet (*n* = 1), alcohol consumption (*n* = 1), late night eating (*n* = 1), unwillingness to modify lifestyle (*n* = 1)

The majority of HCPs (80.72%) reported non-adherence to PPIs. According to HCPs, the most common causes for non-adherence to PPIs were stopping treatment once symptoms improved (53.01%), followed by cost (39.56%), incomplete disease awareness (29.92%), pill burden (23.90%), forgetting to take medication (19.28%), meal-associated dosing (11.04%), and inadequate symptom relief (9.44%).

With regards to the safety aspects, 42.77% HCPs reported no side effects with the use of PPIs. Amongst those who reported side-effects with PPI use, micronutrient deficiency (47.72%) was the most common side effect observed, followed by diarrhea (39.30%), nausea/ vomiting (25.61%), and headache (24.56%). The main concerns regarding the long-term use of PPIs included bone fracture (47.55%), severe kidney problems (37.77%), gastroenteritis (30.16%), *Clostridium difficile* infection (30.16%), dementia (13.59%), and insomnia (7.88%). However, 26.10% of HCPs reported no concerns of long-term side effects (Table 5).

**Table 5:** Side effects of PPIs

Parameters	Number of physicians (*N* = 498)
Common side effects with the use of PPIs	[*n* = 285]
Micronutrient deficiency	136 (47.72)
Diarrhea	112 (39.30)
Nausea/ vomiting	73 (25.61)
Headache	70 (24.56)
Others*	8 (2.81)
Side effects from long term use of PPIs	[*n* = 368]
Bone fracture	175 (47.55)
Severe kidney problems	139 (37.77)
Gastroenteritis	111 (30.16)
Clostridium difficile infection	111 (30.16)
Dementia	50 (13.59)
Insomnia	29 (7.88)
Others^#^	10 (2.72)

Data presented as n (%)

*GERD *gastroesophageal reflux disease, *PPI *proton pump inhibitor

*Others: Anemia (*n* = 1), chronic kidney disease (*n* = 1), constipation (*n* = 1), dryness of mouth (*n* = 2), habit forming (*n* = 1), indigestion (*n* = 1), bowel irregularities (*n* = 1)

^**#**^Others: Micronutrients deficiency (*n* = 2), achlorhydria (*n* = 1), anemia (*n* = 1), gastric polyps (*n* = 1), gastrinoma (*n* = 1), micronutrients deficiency (*n* = 1), cardiovascular disease (*n* = 2), rebound hyperacidity (*n* = 1), severe leg pain (*n* = 1)

According to HCPs, the most preferred lifestyle changes for patients with nocturnal GERD were eating smaller and more frequent meals (72.69%), not lying down for at least two hours after a meal (71.08%), reducing excess weight (66.06%), quitting smoking (65.06%), and keeping the head-end elevated while sleeping (53.61%).

Emphasizing the need for better awareness of nocturnal symptoms of GERD at the patient level, most HCPs (67.47%) preferred in-person training (facilitated by a paramedic or nurse) as the first choice to educate patients on GERD. Moreover, the HCPs preferred digital learning materials (34.54%), videos (42.37%), and printed handouts (46.79%) as their second, third, and fourth choices, respectively, for patient education.

## Discussion

Nocturnal GERD is increasingly recognized as a significant subset of the GERD spectrum. Evidence suggests that individuals experiencing nocturnal reflux not only experience a poorer overall quality of life compared to those with daytime-only reflux but also suffer from disrupted sleep, increased daytime fatigue, and reduced work productivity [15]. This survey offered comprehensive insights into HCPs’ perceptions regarding the disease burden, patient profile, diagnosis, and management of nocturnal GERD in India. The findings provide valuable real-world insights that may help identify gaps between guideline recommendations and clinical practice, thereby informing future research and educational efforts.

A higher prevalence of nocturnal GERD was reported by HCPs in this survey amongst males. Similarly, a study by Mishra et al. reported that out of 60 patients with nocturnal GERD, the majority of patients were males (61.7%) compared to females (38.3%) [16]. In contrast, Shaker et al. reported an overall prevalence of nocturnal GERD symptoms at 13%, with women exhibiting a higher prevalence of GERD symptoms than men (56.0% vs. 44.0%) [4].

Various cross-sectional studies have identified smoking as a significant independent risk factor for GERD [17–19]. A meta-analysis showed that the current smokers had higher odds of developing GERD compared to ex- and non-smokers. However, the odds ratio (OR) between current smokers and both ex- and non-smokers was not statistically significant (OR = 1.04, *P* = 0.065) [20]. Another study indicated that patients with > 20 pack-years had a significantly higher risk of GERD (OR: 1.4; 95% CI: 1.2–1.6). Additionally, current smokers had 23% higher odds of developing GERD compared to non-smokers [21].

Alcohol is frequently recognized as a contributing factor in patients with GERD, particularly nocturnal GERD [22, 23]. Both nicotine and alcohol worsen GERD-related symptoms [23]. Several randomized and cross-sectional studies have indicated a higher prevalence of symptomatic reflux among alcohol consumers [24–26]. These findings align with the present survey, reinforcing the relationship between smoking, alcohol consumption, and GERD risk.

A previous prospective study by Rai V, et al., demonstrated that consumption of fatty food was significantly associated with the prevalence of GERD [27]. Similarly, Kumar et al., identified high intake of spicy and fatty foods (61.5%) and high-stress levels (76.9%) as risk factors strongly associated with GERD [25]. Daytime sleepiness, insomnia, hypertension, and asthma were strong predictors of nighttime heartburn [28]. The present survey also suggested that insomnia, fatty foods, and high-stress levels were major contributors to the weakening of the lower esophageal sphincter, thereby exacerbating GERD symptoms.

Furthermore, late-night meals, high BMI (> 25 kg/m²), hiatal hernia, and long-term and severe heartburn/regurgitation were identified in the RELIEF survey as common risk factors associated with nocturnal heartburn in patients with GERD, align with prior research demonstrating that prolonging the interval between dinner and bedtime effectively reduces the risk and severity of nocturnal GERD [29]. The guidelines suggest avoiding meals before 2–3 h of bedtime, avoid trigger food, elevating head for nighttime GERD symptoms [30]. Obesity is a significant independent risk factor for GERD and hiatal hernia. The probability of a hiatal hernia increases with BMI [31]. More than half of the patient population (58.8%) endured heartburn for over 5 years and were more likely to have a hiatal hernia (51.4%) and higher BMI (29.83%). A multicenter study involving 1,178,274 GERD patients, demonstrated a positive association between higher BMI and GERD. This study also showed that any increase in BMI was associated with an increase in the risk of GERD [32]. Therefore, physicians should recognize obesity and hiatal hernia as critical factors in GERD management, emphasizing the importance of weight reduction strategies as part of comprehensive patient care.

Non-erosive reflux disease is the most common phenotype of GERD, affecting 60–70% of patients, followed by erosive esophagitis and Barrett’s esophagus, which occur in 30% and 6–12% of patients, respectively [33]. A multicenter study that enrolled 396 GERD patients found that 51.3% of patients had non-erosive reflux disease, while 48.7% had erosive esophagitis [17]. The present survey also showed corroborating findings that non-erosive reflux disease was the most common spectrum of GERD, followed by erosive esophagitis and Barrett’s oesophagus. Evidence suggests that NERD patients experience a higher frequency of nocturnal GERD-related symptoms compared to EE patients, as demonstrated in a Korean population study, where 32.8% of NERD patients had a higher risk of nocturnal symptoms and obstructive sleep apnea, compared to 20.9% among EE patients [34]. These findings are further supported by the RELIEF survey, which also confirmed a significant nocturnal symptom burden in GERD patients.

Obesity was the most common comorbidity reported by 79.52% of physicians, followed by diabetes (47.79%), sleep apnea (37.15%), depression (30.12%), and hypertension (24.50%). National Sample Cohort study highlighted that thyroid disease (5.48%), diabetes mellitus (4.44%), dyslipidemia (0.04%), and asthma (9.86%) were prevalent among patients with GERD [35]. On a parallel line, obstructive sleep apnea, hypercholesterolemia, and hypertension have been identified as strong predictors of chronicity of GERD symptoms [36]. The interplay of various comorbidities with GERD underscores the need for a comprehensive approach in managing patients with this condition. Several epidemiologic studies have shown the prevalence of nighttime heartburn and its strong association with sleep disturbances. These disruptions can negatively impact daytime performance, patients’ quality of life, and overall health [37, 38]. Fass R, et al., and Gerson LB, et al., reported that up to 25% of the general population experiences night-time heartburn, with a striking prevalence of up to 75% among those with at least weekly GERD symptoms [39, 40]. The earlier (Shaker et al. 2003) published GALLUP Survey, an US national population-based telephone interview, suggested that night-time heartburn occurs in more than 70% of adults with GERD, leading to sleeping difficulties, impaired next-day function, and significantly worsened health-related quality of life [4]. These findings underscore the significant impact of nocturnal symptoms on sleep quality, daily functioning, and overall health, particularly in individuals with GERD.

The diagnosis of GERD is made solely based on presenting symptoms or in combination with other factors such as responsiveness to antisecretory therapy, esophagogastroduodenoscopy, and ambulatory reflux monitoring [41]. Similarly, in the present survey, most of the HCPs (83.13%) relied on symptoms and medical history as the primary approach to diagnose nocturnal GERD. In comparison, 68.07% relied on response to empiric PPI therapy.

Esomeprazole has also been shown to be more effective in maintaining healing of erosive GERD compared to lansoprazole or pantoprazole [42]. In the present survey, HCPs preferred esomeprazole for treating nocturnal GERD, with 61–80% of patients experiencing symptomatic relief. Corresponding to the present survey, data from clinical trials and meta-analysis indicate that esomeprazole 40 mg OD for up to 8 weeks provides higher rates of healing of erosive GERD and a greater proportion of patients with sustained resolution of heartburn than omeprazole 20 mg, lansoprazole 30 mg, or pantoprazole 40 mg OD [42]. Vcev A et al. reported healing rates of 77.8% with esomeprazole and 72.2% with pantoprazole after 4 weeks. However, at 8 weeks, healing was comparable between esomeprazole and pantoprazole groups (92.2% and. 91.1%). The proportion of heartburn-free days was also similar between the two treatments [43, 44]. Similarly, a pilot study by Chand N, et al. reported that patients treated with esomeprazole 40 mg/day for 4 and 8 weeks showed significant improvements in sleep efficiency, as indicated by wrist actigraphy, and better scores on the Pittsburgh Sleep Quality Index and GERD symptom questionnaires [45]. Furthermore, evidence from a randomized controlled trial demonstrated that esomeprazole 40 mg significantly improved nocturnal GERD symptoms, with 53.1% of patients achieving relief from nighttime heartburn compared to 12.7% with placebo, and also showed significant improvements in sleep quality and daily functioning [46]. These findings suggest that esomeprazole shows a clear advantage in the early phases of treatment, making it a preferred choice over omeprazole, lansoprazole, or pantoprazole for rapid symptom relief and effective management of GERD. However, clinical trial evidence has demonstrated that a potassium-competitive acid blocker (P-CAB), vonoprazan, can rapidly inhibit nighttime acid compared to PPIs [47] Since the PCABs were relatively new in the Indian market, at the time of survey initiation, their adoption in clinical practice was comparatively limited.

In the present survey, HCPs preferred single-dose PPI as the primary treatment for nocturnal GERD, followed by double-dose PPI. The PPIs have been the main treatment for GERD. Guidelines recommend administration of PPI 30–60 min before a meal rather than at bedtime [30].

Although the majority of HCPs reported no side effects with the use of PPIs, micronutrient deficiency was the most commonly reported side effect, followed by diarrhea, nausea/ vomiting, and headache. In line with the present survey, Force RW et al. reported that patients initiating vitamin B12 therapy were more likely to be receiving long-term acid suppression therapy, including PPIs or H2-blockers [48]. Although PPIs are generally well tolerated and considered to have minimal adverse effects, short-term use may cause mild symptoms such as headache, rash, dizziness, and gastrointestinal issues, including nausea, abdominal pain, flatulence, constipation, and diarrhea [49]. However, the long-term use of PPIs has raised concerns due to potential adverse effects, such as calcium and magnesium malabsorption, vitamin B12 deficiency, *Clostridium difficile*-associated disease, and community-acquired pneumonia [50, 51].

The majority of HCPs (62.45%) reported that 1–5% of patients with nocturnal GERD eventually required surgical management, while 21.69% indicated that 6–10% of patients needed surgical intervention.

The survey highlights the pressing need for greater awareness and sensitization at the clinic level regarding nocturnal GERD. Many HCPs emphasized the importance of in-person training sessions facilitated by paramedics to educate patients about the condition. Additionally, digital learning materials, videos, and printed handouts were preferred as effective supplementary educational tools to enhance patient understanding and adherence to treatment.

### Limitations

Despite the limited sample size, this survey provides valuable insights into HCPs ' perceptions and practices regarding nocturnal GERD management. However, certain limitations should be acknowledged. Responses were based on physicians’ perceptions and practices, susceptible to self-reporting errors or subjective interpretations. As is the case with most questionnaire-based surveys, there is a possibility of recall bias. The study employed a convenience sampling strategy that focused on gastroenterologists with an academic interest in managing Nocturnal Gerd. This may have introduced selection bias toward early adopters and enthusiasts of PPIs. The findings should be interpreted as exploratory insights rather than representative of national practice pattern. The survey focused solely on HCPs’ perceptions, overlooking patient perspectives in nocturnal GERD management. Therefore, the results may not accurately represent epidemiological trends or clinical outcomes but instead provide insights into real-world management approaches and challenges encountered in routine practice. Consequently, the study did not assess the clinical effectiveness of combination therapies such as PPIs with alginates and/or H2 receptor antagonists. The lack of systematically captured endoscopy data represents a limitation of the survey.

## Conclusion

Although published data exist on the prevalence of GERD in general, data specifically addressing nocturnal reflux from India are limited. The RELIEF Survey provided real-world insights into HCPs’ perspectives towards management of nocturnal GERD in India. HCPs reported nocturnal symptoms like heartburn and regurgitation in nearly one-fourth of their GERD patients, emphasizing the need for lifestyle modifications and suitable pharmacological interventions. The standard approach for managing nocturnal GERD involves PPI therapy, with esomeprazole being the preferred choice for achieving symptomatic relief. This survey underscores the importance of increasing awareness of nocturnal GERD and adherence to PPIs to improve long-term patient outcomes.

## Supplementary information

Supplementary Material 1.

## Data Availability

Deidentified data will be shared upon reasonable request from the corresponding author.
