# Supplementary material for: Real world clinical practice patterns in nocturnal GERD management: insights from nationwide RELIEF survey
Source: BMC Gastroenterol. 2026 Jul 23;26:670. doi: 10.1186/s12876-026-05084-x (PMC13681958; doi:10.1186/s12876-026-05084-x)
Supplement: Supplementary file 1 [file 12876_2026_5084_MOESM1_ESM.docx]

**Survey Questionnaire**

**Version No.:01**

- Please tick only one appropriate option per question, unless specified for that question
- Please answer the questions below, considering your practice

**Q1. In your clinical practice, what percentage of patients** **with GERD do you encounter in a month? Please select one option only.**

A. ≥10
B. 11-20
C. 21-30
D. >30

**Q2. What percentage of patients diagnosed with GERD complain of nighttime symptoms (heartburn and regurgitation)? Please select one option only.**

A. 0-20%
B. 21-30%
C. 31-40%
D. 41-50%
E. 51-60%
F. 61-70%
G. 71-80%
H. >80%

**Q3. In your clinical practice, what is the gender-wise prevalence observed for nocturnal GERD? Please select one option only.**

A. Majority male
B. Majority female
C. Equal

**Q4.** **Which of the following risk factors do you see commonly in patients with nocturnal GERD? Please tick relevant options.**

A. Late night meals
B. Smoking
C. Equal
D. BMI >25 kg/m^2^
E. High-fat diet
F. Drinking
G. Hiatal hernia
H. Long term and severe heartburn/ regurgitation
I. Others

Q5. **Which GERD patient group is most frequently associated with nocturnal symptoms in your practice? Please select one option only.**

A. Non-erosive reflux disease
B. Erosive esophagitis
C. Barrett’s esophagus

**Q6. Which of the following co-morbidities are commonly seen in your patients with nocturnal GERD? Please tick relevant options.**

A. Obesity
B. Diabetes
C. Sleep apnea
D. Depression
E. Hypertension
F. COPD
G. Coronary heart disease
H. Arthritis
I. Others

**Q7. Which of the following do you rely on to** **diagnose nocturnal GERD in most patients? Please rank stepwise, 1 being performed first and 5 being last.**

A. Symptoms and medical history

B. Response to empiric PPI therapy

C. Esophageal manometry

D. Endoscopy and biopsy

E. Esophageal impedance-pH monitoring

**Q8. Which lifestyle recommendations in your experience commonly help nocturnal GERD patients? Please tick relevant options.**

A. Eating smaller and more frequent meals each day

B. Not lying down for at least two hours after a meal

C. Reducing excess weight

D. Quitting smoking

E. Keeping head elevated while sleeping

F. Others

**Q9. Which aspect do you feel is important while choosing a PPI? Please rate accordingly with 1 being most important and 7 being the least.**

A. Cost

B. Comorbidity

C. Minimum or no drug-drug interactions

D. Safety concerns

E. Efficacy

F. Others

**Q10. Please rate the following PPIs according to your preference for managing nocturnal GERD with 1 being most preferred and 6 being the least.**

A. Dexlansoprazole

B. Esomeprazole

C. Lansoprazole

D. Omeprazole

E. Pantoprazole

F. Rabeprazole

**Q11. What treatment regimen do you recommend most commonly for patients with nocturnal GERD? Please select one option only.**

A. Single dose PPI (e.g., 40 mg esomeprazole once daily)

B. Double dose PPI (e.g., 40 mg esomeprazole twice daily)

C. Split dose PPI + alginate

D. Single dose PPI + H2RA

E. Split dose PPI (e.g., 20 mg esomeprazole twice daily)

F. Split dose PPI + H2RA

G. Others

**Q12. In your clinical practice, what is the mean duration of therapy to achieve resolution of nocturnal GERD symptoms with PPIs? Please select one option only.**

A. <4 weeks
B. 4-8 weeks
C. >8 weeks

**Q13.** **In your clinical practice what is the mean duration of maintenance therapy to achieve resolution of nocturnal GERD symptoms with PPIs? Please select one option only.**

A. <3 months
B. 3-6 months
C. >6 months-<1 year
D. ≥1 year

**Q14. Amongst the patients in whom you have prescribed esomeprazole, approximately how many patients achieved symptomatic relief? Please select one option only.**

A. 20-40%
B. 41-60%
C. 61-80%
D. >80%

**Q15. In your clinical practice what are the advantages of esomeprazole you find relevant for clinical response? Please tick relevant options.**

A. Provides greater acid control
B. Maintains pH >4 for 16.8 h
C. Relieves night -time heartburn and sleep disturbance symptoms
D. Improves healing rate of the esophageal mucosa
E. Higher systemic bioavailability
F. All of the above

**Q16.** **What is the rate of recurrence of nocturnal GERD after completion of (3-6 months) PPI treatment? Please select one option only.**

A. <20%
B. 21-40%
C. 41-60%
D. >60%

**Q17. What percentage of patients report refractory GERD symptoms? Please select one option only.**

A. <20%
B. 20-30%
C. 41-50%
D. 51-60%
E. >60%

**Q18.** **What are the common reasons for refractory GERD in your practice? Please tick relevant options.**

A. Non-adherence
B. Comorbidities
C. Inadequate dosage
D. Incorrect diagnosis
E. Others

**Q19. What percentage of nocturnal GERD patients eventually require surgical management? Please select one option only.**

A. 1-5%
B. 6-10%
C. 11-20%
D. 21-40%
E. 41-50%

**Q20. In your clinical practice do you observe non-adherence to PPIs?**

A. Yes
B. No

**Q21. Which are the common causes that you have observed for non-adherence to PPIs? (You may tick up to 3 options for this question)**

A. Stopped treatment once symptoms improved
B. Cost
C. Incomplete disease awareness
D. Pill burden
E. Forget to take
F. Meal-associated dosing
G. Inadequate symptom relief.

**Q22. Which side effects do you commonly observe in your clinical practice with PPI?**

A. No side effects
B. Micronutrient deficiency
C. Diarrhea
D. Nausea/ vomiting
E. Headache
F. Others

**Q23.** **Which side effects are you concerned about due to long term use of PPI?**

A. Bone fracture
B. Severe kidney problems
C. No side effects
D. Gastroenteritis
E. *Clostridioides difficile* infection
F. Dementia
G. Insomnia
H. Others

**Q24. In your practice, which is the preferred way to educate your patients? Please rate according to your preference with 1 being most preferred and 4 being the least.**

A. Digital learning material
B. In-person training (paramedic or nurse)
C. Printed handouts
D. Video
